# Graphene and the Immune System: A Romance of Many Dimensions

**DOI:** 10.3389/fimmu.2017.00673

**Published:** 2017-06-13

**Authors:** Sourav P. Mukherjee, Massimo Bottini, Bengt Fadeel

**Affiliations:** ^1^Nanosafety and Nanomedicine Laboratory, Division of Molecular Toxicology, Institute of Environmental Medicine, Karolinska Institutet, Stockholm, Sweden; ^2^Department of Experimental Medicine and Surgery, University of Rome ‘Tor Vergata’, Rome, Italy; ^3^Sanford Burnham Prebys Medical Discovery Institute, La Jolla, CA, United States

**Keywords:** graphene, macrophage, endotoxin, inflammasome, pattern recognition receptors

## Abstract

Graphene-based materials (GBMs) are emerging as attractive materials for biomedical applications. Understanding how these materials are perceived by and interact with the immune system is of fundamental importance. Phagocytosis is a major mechanism deployed by the immune system to remove pathogens, particles, and cellular debris. Here, we discuss recent studies on the interactions of GBMs with different phagocytic cells, including macrophages, neutrophils, and dendritic cells. The importance of assessing GBMs for endotoxin contamination is discussed as this may skew results. We also explore the role of the bio-corona for interactions of GBMs with immune cells. Finally, we highlight recent evidence for direct plasma membrane interactions of GBMs.

O brave new worlds, that have such people in them!

Edwin A. Abbott, *Flatland*. *A Romance of Many Dimensions* (1884).

## Introduction

Graphene and its derivatives have attracted considerable attention for various applications in science and technology (1, 2). Graphene oxide (GO), in particular, is being intensively investigated for various biomedical applications including drug delivery and bioimaging, and as biosensors (3). GO offers interesting physicochemical properties including its large surface area, ease of surface functionalization, and superior colloidal stability in aqueous media (4). However, increasing production and use of graphene-based materials (GBMs) also necessitates careful scrutiny of the impact of such materials on cells and tissues (5). Understanding the interactions with the immune system is of particular importance (6). Once inside the body, a foreign material will encounter phagocytic cells of the innate immune system, such as neutrophils, macrophages, and dendritic cells (DCs). These cells represent the first line of defense against foreign intrusion (microorganisms, particles), and they also clear cell debris, thus playing an important role in tissue homeostasis. Macrophages are involved in the initiation, propagation, and resolution of inflammation (7), while DCs are antigen-presenting cells that act as a bridge between the innate and adaptive arms of the immune system. Neutrophils are specialized in killing bacteria and other microorganisms, although recent studies have suggested that these cells may also orchestrate adaptive immune responses (8). It is important to note that macrophages that reside in different tissues are not only important effectors of the innate immune response but may also contribute to acute or chronic tissue injury resulting from toxicant exposure through the release of a host of soluble mediators, e.g., reactive oxygen or reactive nitrogen species, proteolytic enzymes, and pro-inflammatory cytokines or chemokines (9). Thus, as emphasized before by Laskin et al. (9), macrophages are mediators of both “defense and destruction.”

**Figure 1:**
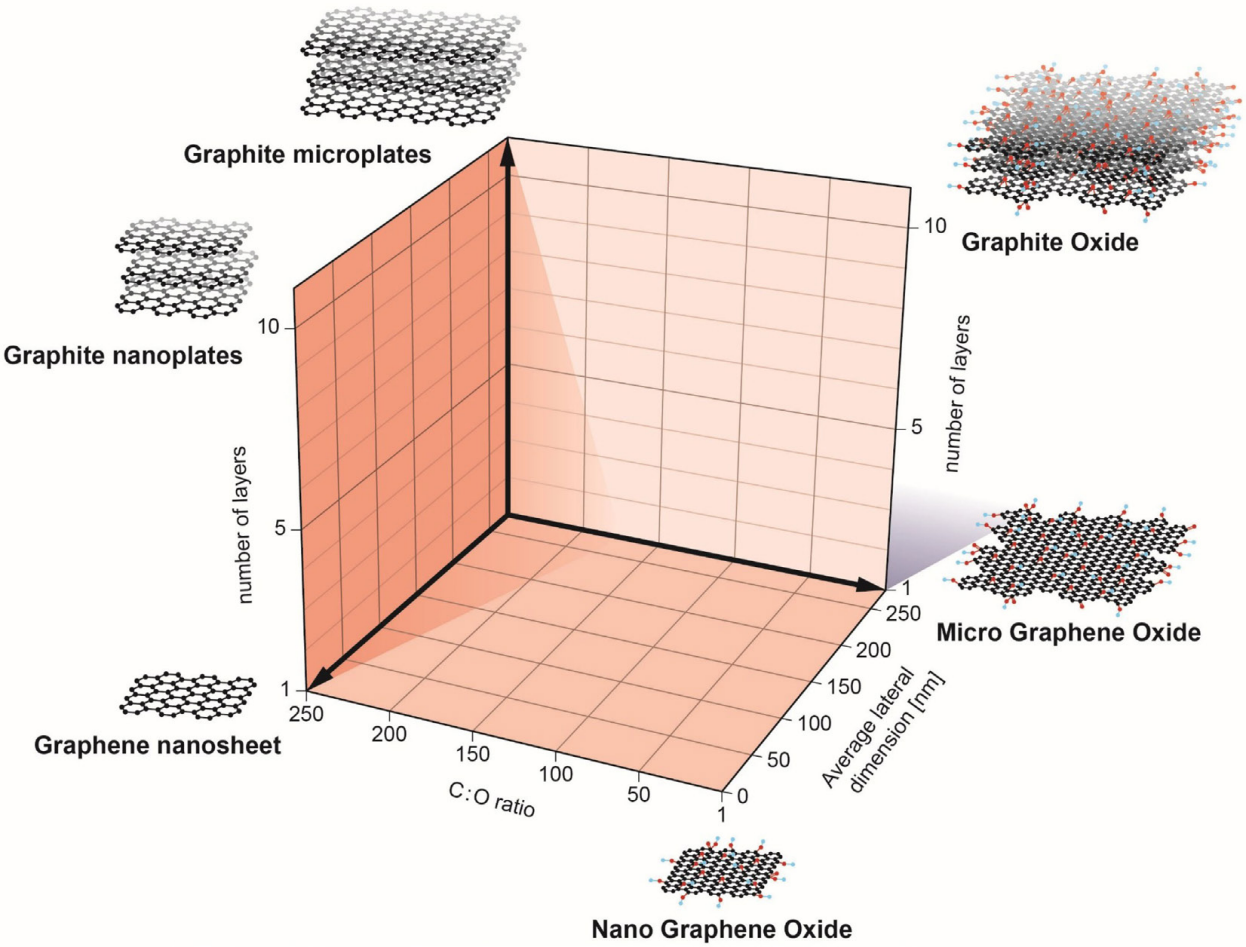
Classification of graphene-based materials (GBM). In the European Commission funded GRAPHENE Flagship project, three physicochemical descriptors were defined to enable the classification of GBMs: number of graphene layers, average lateral dimension, and atomic carbon/oxygen ratio. The proposed classification framework will help to determine the role of specific physicochemical properties on the health and safety profile of GBMs. Reproduced from: Wick et al. (10) with permission from John Wiley & Sons, Inc.

Recently, a classification system (Figure 1) was proposed by researchers in the EU-funded GRAPHENE Flagship Project as a starting point for the categorization of distinct graphene types (10). In brief, three physicochemical properties of GBMs were highlighted: (i) the number of graphene layers, (ii) the average lateral dimensions, and (iii) the carbon-to-oxygen (C/O) atomic ratio; the inclusion of the C/O ratio as a functional property can be justified by the fact that GBMs are both structurally and chemically heterogeneous. Indeed, as stated by Wick et al. (10) different members of the GBM family do not share the same “standard” surface. The surface of pristine graphene is hydrophobic while in the case of GO, surfaces consist of hydrophobic islands interspersed with hydrophilic regions. This could potentially influence the interactions of these materials with biological systems. Here, we discuss recent studies on the interaction of GBMs with cells of the innate immune system, including macrophages, neutrophils, and DCs. Notably, while these cells all share the propensity for phagocytosis, we also explore emerging evidence that GBMs may exert direct effects on the plasma membrane of immune cells in the absence of cellular uptake. The biodegradation of carbon-based materials by immune cells including neutrophils and macrophages has been highlighted in other recent review articles (3, 11, 12) and is not discussed here. We will mainly focus our discussion on studies using macrophages or macrophage-like cell lines as there are few studies to date on GBM effects on neutrophils and DCs. Nevertheless, as more and more studies are emerging, we may begin to understand how the immune system responds to 2D objects—a journey into flatland.

## The Importance of Endotoxin Assessment

Endotoxins, also known as lipopolysaccharides (LPS), are large (200–1,000 kDa), hydrophobic, heat-stable molecules that form part of the outer membrane of gram negative bacteria (13). LPS is a potent inflammatory mediator which activates immune cells *via* pattern recognition receptors leading to the secretion of pro-inflammatory mediators, e.g., tumor necrosis factor (TNF)-α, and interleukin (IL)-1β (14). As many nanomaterial-enabled drug carriers or diagnostic devices are engineered to target the immune system (or to avoid interactions with it), it is increasingly important to understand immune response to these materials (15, 16). Of particular importance in this context is the fact that nano-biomaterials and pharmaceutical products alike are commonly contaminated with endotoxins which could lead to septic shock and organ failure if administered to patients (17). Endotoxin detection in pharmaceutical products is performed using two different methods. The rabbit pyrogen test (RPT) enables the detection of pyrogens in general by measurement of fever development after injection of the test sample; it is expensive and requires the use of large numbers of animals (18). The second type of endotoxin detection method, the *Limulus* amebocyte lysate (LAL) assay, is based on the blood of wild horseshoe crab populations. While the RPT assay can only detect the presence of endotoxins indirectly, the LAL assay is more specific to endotoxins as it takes advantage of the LPS-sensitive serine protease Factor C. Upon activation, Factor C induces a coagulation cascade leading to the amplification of the LPS stimulus and the formation of a firm gel clot. All LAL assays are in principle based on this coagulation cascade, but they have been further modified to enable quantitative determination of endotoxins (18). Both of these tests have a long history of use for traditional pharmaceuticals and medical devices and are routinely used in drug development. More recently, the recombinant factor C (rFC) assay and the macrophage activation test (MAT) were recognized as alternatives to the LAL assay. The MAT, which mimics the human fever reaction, was established as an alternative test for pyrogen testing (19). Importantly, the European Directive 2010/63/EU on the protection of animals used for scientific purposes enforces the replacement of animal tests when validated alternatives exist. While the LAL assay is known to be very sensitive, several laboratories have reported problems of interference of various types of nanomaterials with one or more of the LAL assay formats (20–22). Indeed, carbon-based nanomaterials including GBMs were shown to interfere with the LAL assay, which may lead to erroneous results or mask the effects of the materials themselves (23, 24). In a recent study, the authors suggested that repeated cycles of autoclaving may reduce the endotoxin content of carbon-based nanomaterials including pristine graphene and that the native versus depyrogenated materials elicited distinct macrophage responses *in vitro* (25). However, the chromogenic LAL assay was employed to assess for endotoxin contamination, calling into question whether the proposed depyrogenation procedure worked (25). TLR4 reporter cells were suggested as an alternative assay to evaluate endotoxin contamination of metal/metal oxide nanoparticles (21). However, recent work has implied that GO could trigger cell death in macrophages *via* TLR4 (discussed below), meaning that the use of such reporter cells could also yield ambiguous results. Mukherjee et al. (23) developed a novel assay for endotoxin detection to circumvent problems with assay interferences of GBMs. The assay, designated the TNF-α expression test (TET), is based on the detection of TNF-α secretion in primary human monocyte-derived macrophages incubated in the presence or absence of a specific endotoxin inhibitor. It was shown that when non-cytotoxic doses of GBMs were applied, the TET enabled unequivocal detection of LPS with a sensitivity that was comparable to the LAL assay. Guidelines for the preparation of endotoxin-free GO were also presented (23).

## Bio-Corona Formation: Shelter from the Storm

When a nanomaterial is introduced into a living system it interacts with biological molecules (proteins, lipids, etc.) leading to the formation of a so-called bio-corona on the surface (26), or, to put this in immunological terms, the nanomaterial is opsonized (the process whereby pathogens or cells are rendered more susceptible to phagocytosis). Detailed studies of various types of nanoparticles have shown that bio-corona formation depends not only on the size or surface curvature of the particle but also on surface properties such as the degree of hydrophobicity (27–29). The bio-corona has been shown to modulate cellular uptake of nanomaterials (30), and a recent study suggested that proteins present in the original protein corona are retained on the nanoparticles until they reach the lysosomes (31). Moreover, the adsorption of proteins may mitigate the cytotoxic effects of nanomaterials. Indeed, *in vitro* studies have shown that the adsorption of serum proteins reduces the cytotoxicity of carbon nanotubes (CNTs) (32) as well as GO (33), and based on a combination of experimental and theoretical approaches, it was suggested that the bio-corona mitigates the cytotoxicity of GO by limiting its penetration into the cell membrane (34). Furthermore, modeling studies suggested that graphene, due to its hydrophobic nature, may interrupt hydrophobic protein–protein interactions (35). Indeed, it is important to recognize the differences in physicochemical properties between different members of the GBM family, not least with respect to the potential interaction with proteins. Graphene is essentially a single atomically thin sheet of sp^2^-bonded carbon atoms, whereas GO is an oxidized graphene sheet derivatized by carbonyl and carboxyl groups at the edges and displaying epoxide and hydroxyl groups on the basal plane (36). Moreover, graphene and GO have different surface energies—an important parameter affecting dispersibility. Thus, graphene is hydrophobic and dispersible in organic solvents whereas GO can be dispersed in water (37). The latter property derives mostly from the ionizable edge carboxyl groups, the basal plane being essentially a network of hydrophobic islands of unoxidized benzene rings surrounded by polar groups (38). Additionally, small GO sheets are more hydrophilic than larger ones because of greater charge density, which could impact on bio-interactions.

Intravenously injected nanomaterials can adsorb a wide range of proteins in the blood (39). The bio-corona of blood proteins is rapidly formed, and it has been shown to affect hemolysis and thrombocyte activation (40). Furthermore, complement activation on the surface of nanomaterials is of particular concern when it comes to clinical applications. In fact, complement proteins have been consistently identified in or on nanoparticle coronas (28, 30, 40, 41). The complement system is a critical component of the innate immunity in the blood; it is a proteolytic cascade typically triggered *via* three distinct pathways (classical, lectin, and alternative) that converge to generate the same set of effector molecules at the third component of complement (C3) (42). Complement proteins opsonize pathogens and cells for engulfment *via* complement receptors and could conceivably promote nanomaterial uptake as well. However, certain complement factors may instead confer “stealth” properties to nanomaterials by preventing further complement activation, as shown in a recent study on GO (43). Complement activation also liberates two potent effector molecules (C3a and C5a) that play important roles in the recruitment and activation of inflammatory cells as well as anaphylaxis, a serious allergic reaction that is rapid in onset and may cause death (44). Several reports have documented pathway-specific complement activation by various types of nanomaterials including carbon-based nanomaterials such as CNTs (45, 46) and GO (47, 48). The question is: could particle surfaces be engineered to avoid protein adsorption and/or unscheduled complement activation? The attachment of polymers such as poly(ethylene glycol) (PEG) on particle surfaces is a common approach in nanomedicine, and the traditional view has held that PEGylation completely prevents protein adsorption, thereby preventing the clearance of particles by the reticuloendothelial system. However, if this were true, then how does one explain complement activation on PEGylated particles? In recent years, the view has emerged that PEGylation of nanomaterials only partially blocks protein adsorption and may even promote the formation of a bio-corona that is distinct in comparison to the corona formed on pristine nanomaterials (49, 50). Indeed, in a recent study using macrophage-like RAW264.7 cells, the adsorption of specific proteins was shown to be required to prevent uptake of PEG- or poly(ethyl ethylene phosphate) (PEEP)-coated polystyrene particles (51).

The choice of polymer coating matters. Luo et al. (52) reported that PEG-coating prevented uptake of GO by murine peritoneal macrophages while coating with cationic poly(ether imide) (PEI) favored uptake at low doses, but compromised cell viability at high doses. In another recent study, the authors provided evidence that PEGylated GO of approximately 200 nm in lateral size induced immune responses (cytokine release) in murine peritoneal macrophages; interestingly, comparable levels of activation were also observed following PEGylation of the non-carbon-based 2D material, molybdenum-disulfide (MoS_2_) (53). The authors speculated that integrin signaling could account for the enhanced cytokine responses in cells exposed to PEG-GO. Overall, the study suggested that PEGylation does not serve to passivate the surfaces of 2D materials. Xu et al. (54) prepared a series of GO derivatives including aminated GO (GO-NH_2_), poly(acrylamide)-functionalized GO (GO-PAM), poly(acrylic acid)-functionalized GO (GO-PAA), and PEG-functionalized GO (GO-PEG), and compared their toxicity with pristine GO. The GO materials all displayed lateral dimensions in the range of 100–500 nm and the ζ-potential was negative for all the materials in cell culture medium due to protein adsorption. Among these GO derivatives, GO-PEG and GO-PAA induced less toxicity toward murine J774A.1 macrophage-like cells than pristine GO, and GO-PAA proved to be the most biocompatible one, both *in vitro* and in mice (54). The differences in biocompatibility were suggested to be due to differences in the compositions of the bio-corona, especially whether or not immunoglobulin G (IgG) was present; GO-PAA and GO-PEG had less IgG content in their protein coronas (30−40%) than GO, GO-NH_2_, and GO-PAM (50−70%). IgG is a well-known opsonin that plays a key role in the clearance of pathogens. This study points toward strategies for safe design of GO for biomedical applications and underscores the importance of the bio-corona (54).

## Effects on Macrophages: Breaking and Entering

**Figure 2:**
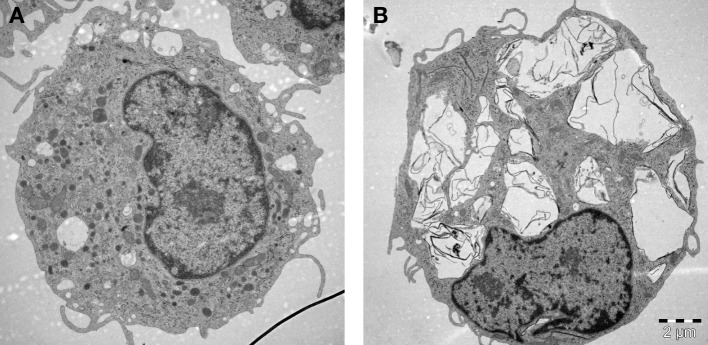
Macrophages are professional phagocytic cells capable of ingesting micron-sized graphene oxide (GO). These TEM images show primary human monocyte-derived macrophages cultured for 24 h in cell medium alone **(A)** or with 10 µg/mL GO **(B)**. The cells readily internalized GO (present in cytoplasmic vesicles) without ultrastructural signs of cell death. Cells were maintained in RPMI-1640 medium supplemented with 10% fetal bovine serum. TEM: Kjell Hultenby, Electron Microscopy Core Facility, Karolinska Institutet.

Macrophages (“big eaters”) are professional phagocytes arising from the bone marrow; these cells are referred to as monocytes when they are present in the peripheral circulation and “macrophages” when they reside in tissues. Macrophage phagocytosis of pathogens is facilitated through opsonization by immunoglobulins and components of the complement system, but engulfment may also be non-specific. We have noted that primary human monocyte-derived macrophages efficiently engulfed GO without signs of acute (24 h) cell death (Figure 2). GO was found in membrane-enclosed vesicles in the cytoplasm, suggesting uptake *via* endocytosis. Other recent studies using macrophage-like THP.1 cells suggested that phagocytosis influences the degree of cytotoxicity of GO to some extent (55). However, while inhibition of phagocytosis blunted the cytotoxicity of single-layer GO, the effects of multi-layered GO were shown to be similar regardless of whether or not phagocytosis occurred. Furthermore, other recent studies have reported that GO sheets with large lateral dimensions could align with the plasma membrane of macrophages (so-called “masking” effect) and it was hypothesized that this parallel arrangement of GO sheets on the cell surface could either promote their internalization, or isolate the cells from their environment, thus compromising cell viability and/or cell function (56). Similarly, Ma et al. (57) reported that large GO sheets showed a stronger “adsorption” to the plasma membrane of murine macrophage-like J774.A1 cells with less phagocytosis, while small GO sheets were more readily taken up by cells. The authors also found that large GO promoted a pro-inflammatory polarization of macrophages both *in vitro* and *in vivo*. In contrast, other investigators have reported that small GO sheets elicited more profound effects on human immune cells (monocytes) when compared to large GO (58). Li et al. (59) suggested, on the basis of experimental and theoretical studies, that micron-sized graphene sheets entered cells through membrane piercing or slicing (“edge-first” uptake). In fact, several studies in recent years using different cell models have suggested that GO could exert direct effects on the plasma membrane of cells, with or without cell death. For instance, micron-sized GO sheets were found to induce the formation of vacuoles in the cytosolic compartment of cells leading to an increased cell membrane permeability for small molecules; this vacuolization was only observed in cells that overexpressed the water channel, aquaporin (AQP1) (60). GO was also shown to compromise plasma membrane and cytoskeletal function in various cell lines without significant signs of cell death, and interactions with integrins in the cell membrane were implicated in this process (61). The authors proposed that this could be exploited to sensitize cancer cells to chemotherapeutic agents, but it was not demonstrated whether these effects were specific for cancer cells. Furthermore, single-layer graphene was found to produce holes (pores) in the membranes of A549 lung carcinoma cells and macrophage-like RAW264.7 cells, leading to a substantial loss of cell viability (62). Pore formation occurred even in the presence of serum, and molecular dynamics simulations suggested that the pore formation was dependent on lipid extraction. Indeed, previous experimental and theoretical studies have suggested that the antibacterial behavior of graphene arises from the formation of pores in the bacterial cell wall (63), possibly due to lipid extraction from bacterial membranes (64). Finally, in another recent study, nano-sized GO sheets were shown to induce membrane ruffling in a variety of different cell lines with concomitant shedding of membrane fragments (65). The underlying mechanism was not disclosed, although changes in the levels of Ca^2+^ in the cell are known to regulate the formation of such actin-driven membrane protrusions. Thus, it appears that GBMs are capable of interacting with cells in a variety of different ways including masking, piercing, ruffling/shedding, pore formation (possibly *via* membrane lipid extraction), and/or internalization into cells. How does one make sense of such disparate observations? First of all, there could be important differences in the test material itself, including the thickness and the lateral dimensions (and, of course, the dose of the material added to cell cultures). Moreover, differences in cell culture conditions (including whether or not the cell culture medium is supplemented with serum) may come into play. Indeed, it has been noted that the composition of the cell culture medium itself could critically affect the way in which GO (and other nanomaterials) interact with cells (66). Finally, the fact that different cell models are used may account for the striking differences in cellular outcomes in the studies reported here. Thus, it is important to understand that transformed cell lines are only a model of normal cells, and that so-called macrophage-like cell lines do not fully recapitulate the behavior of primary macrophages (67). It is also important to realize that there are many different macrophage populations and that the phenotype or activation status of macrophages may affect how these cells respond to nanomaterials, as we and others have recently shown (68, 69). Notwithstanding, the view is emerging that GO could have direct effects on the cell membrane and further studies are needed to understand these interactions. This is obviously important if GBMs are to be used as “smart” carriers of a therapeutic payload to specific cell populations in the body.

## Effects on Macrophages: Inflammasome Activation

Inflammasomes are multiprotein complexes that activate caspase-1, which leads to maturation and secretion of the pro-inflammatory cytokines IL-1β and IL-18 (70). Inflammasome activation is important for host defense and pathogen clearance. In addition, inflammasome activation is implicated in the development of various chronic inflammatory diseases, and the NLRP3 inflammasome is activated by endogenous “danger” signals such as monosodium urate, the causative agent in gout (71), and by cholesterol crystals that are present in atherosclerotic lesions (72). Moreover, an emerging body of literature shows that carbon-based nanomaterials, including long and fiber-like multi-walled CNTs (73, 74) as well as small, spherical carbon nano-onions (75) and hollow carbon spheres (76), are able to activate the inflammasome complex in phagocytic cells (macrophages) with subsequent secretion of IL-1β. GO was recently shown to trigger IL-1β production in myeloid (THP.1) and epithelial (BEAS-2B) cells, respectively (77). We have found that GO of varying lateral dimensions triggered the inflammasome in primary human monocyte-derived macrophages and we noted that cellular uptake of GO was required for IL-1β production (Mukherjee et al., unpublished observations). Similarly, Cho et al. (55) reported that phagocytosis inhibition abolished IL-1β secretion in THP.1 cells exposed to single-layer GO, but not in cells exposed to multi-layered GO. Taken together, a range of carbon-based nanomaterials including not only fiber-like materials but also spherical particles and flat materials such as GO trigger inflammasome activation. Needless to say, it is important to exclude endotoxin contamination of the test material when conducting such experiments as LPS is known to act as a co-signal for inflammasome activation (78). Indeed, endotoxin is often used to stimulate cells *in vitro* when assessing NLRP3 inflammasome activation (73), and this is certainly relevant in the context of a microbial challenge. However, it is pertinent to ask how the inflammasome is activated in sterile (nanomaterial) induced inflammation. In a recent publication, Jessop et al. (79) provided evidence for a role of high-mobility group box 1 (HMGB1) for MWCNT-induced inflammasome activation *in vitro* and *in vivo*. Cholesterol crystals are known to act as “danger” signals and a recent study demonstrated that cholesterol crystals triggered neutrophils to release neutrophil extracellular traps (NETs) (see below) which, in turn, primed macrophages for cytokine release (80). This finding suggests that a “danger” signal may drive sterile inflammation through its interaction with neutrophils. Further studies should address whether the release of HMGB1 or other “danger” signals plays a role in GO-induced inflammasome activation.

Toll-like receptors (TLRs) are so-called pattern recognition receptors that recognize structurally conserved molecules expressed by microbes, leading to the activation of immune responses (81). TLR4, the pattern recognition receptor for LPS (endotoxin), has been suggested to recognize a host of other endogenous factors, ranging from proteins to metal ions. However, direct activation of a single receptor by such a range of molecular signals is difficult to explain from a structural point of view, and care should be taken to exclude potential endotoxin contamination (82). On the other hand, it has been suggested that TLRs might sense the display of hydrophobic patches on a variety of molecules, which may explain the apparent promiscuity of this class of pattern recognition receptors (83). Interestingly, Qu et al. (84) reported that GO with a size of about 1–2 µm induced TLR4-dependent cell death in bone marrow-derived macrophages from mice and presented evidence that this occurred, at least in part, through a paracrine TNFα-dependent mechanism. In previous work, Chen et al. (85) showed that GO induced autophagy and cytokine secretion in a TLR-dependent manner in the mouse macrophage cell line RAW264.7. In contrast, our recent studies have suggested that GO triggers inflammasome activation with secretion of IL-1β in primary human monocyte-derived macrophages without engaging the TLR signaling pathway (Mukherjee et al., unpublished results). Notably, no cell death was observed in macrophages exposed to GO, in marked contrast to the aforementioned studies. Care was taken to control for endotoxin contamination prior to cell exposures. We suggest that endotoxin testing should be mandatory when studying putative interactions of GO with TLRs.

**Figure 3:**
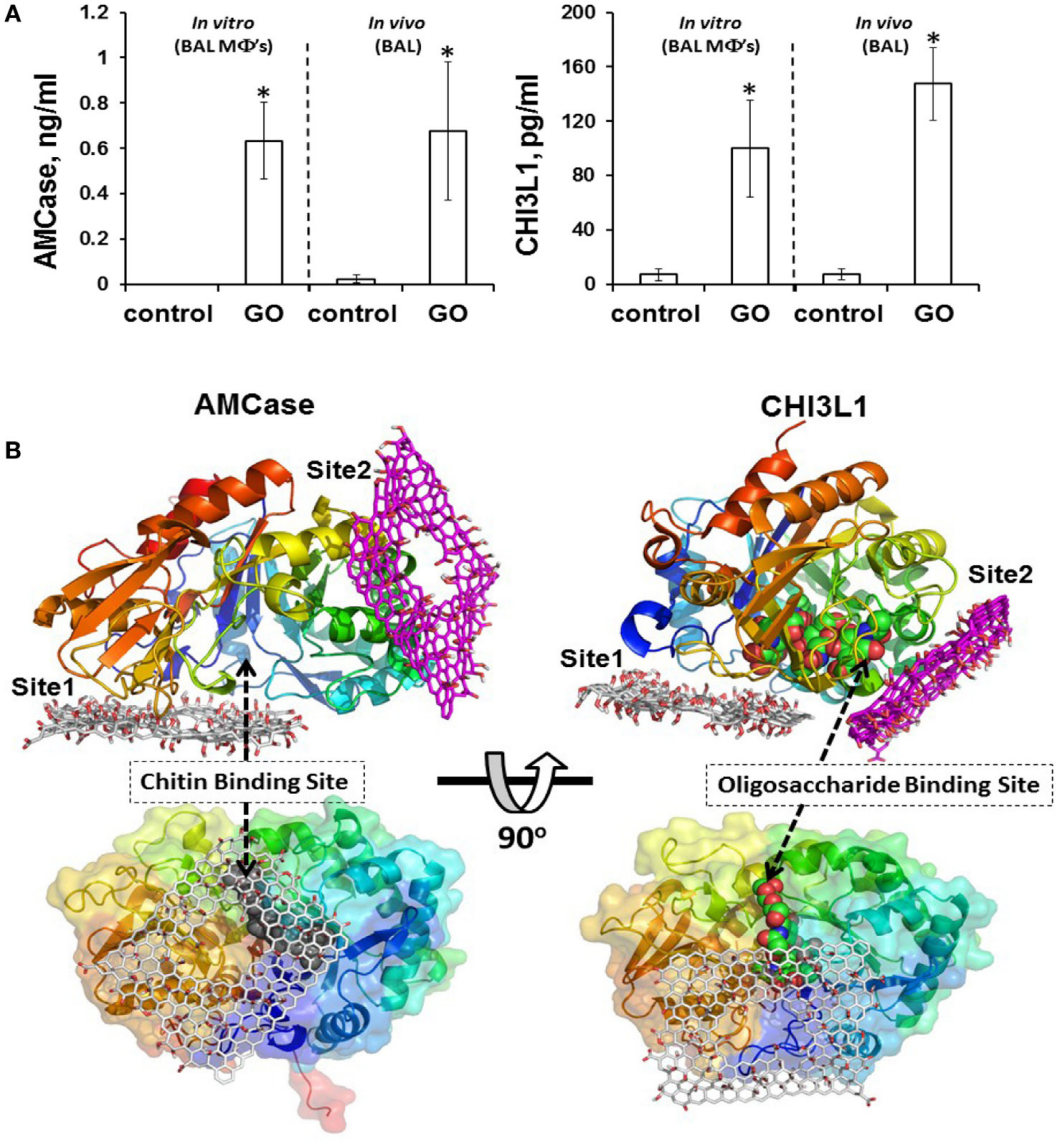
GO triggers macrophage production of the mammalian chitinases, chitinase 3-like 1 (CHI3L1) and acidic mammalian chitinase (AMCase), whose expression is associated with asthma. **(A)** GO stimulates accumulation of AMCase and CHI3L1 in the lungs of mice. Levels of CHI3L1 and AMCase were measured in BAL fluid of mice 7 days after exposure to GO, or in supernatants from cultured macrophages isolated from BAL fluid of mice exposed to GO or vehicle 24 h after exposure. **(B)** Molecular modeling suggests that GO may directly interact with chitinases. The two predicted binding sites of GO, site 1 and site 2, are shown for AMCase and CHI3L1, respectively. The occlusion of the entrance to the chitin binding site in AMCase could lead to inhibition of its activity. Reproduced from: Shurin et al. (86), with permission from The American Chemical Society.

In most of the examples provided here, the impact of GO on isolated macrophages or macrophage-like cells was investigated. While such studies may provide important insights regarding the mode of entry of GO into cells and on the signaling pathways affected following cellular interactions, studies in living organisms are needed to assess the overall response to GO and the interplay between both arms of the immune system. Shurin et al. (86) recently provided a detailed analysis of how exposure to GO modulates the allergic pulmonary response. To this end, the authors used a murine model of ovalbumin (OVA)-induced asthma, and found that GO, given at the sensitization stage, augmented airway hyperresponsiveness (AHR) and airway remodeling, while at the same time, the levels of the Th2 cytokines, IL-4, IL-5, and IL-13 were suppressed in bronchoalveolar lavage (BAL) fluid in exposed mice (86). Moreover, exposure to GO during sensitization with OVA decreased eosinophil accumulation and increased recruitment of macrophages in BAL fluid. Exposure to GO also increased the macrophage production of the mammalian chitinases, chitinase 3-like 1, and AMCase, whose expression is associated with asthma (87), and molecular modeling suggested that GO may directly interact with chitinases, affecting their activity (Figure 3). Taken together, these results indicated that pulmonary exposure to GO initiates a novel mechanism of nanomaterial-induced airway remodeling and AHR in a mouse model of asthma that is independent from eosinophilic airway inflammation and Th2-mediated immune responses, with the possible involvement of mammalian chitinases (86).

## Effects on Neutrophils: Tangled up in Blue

Neutrophils are the most abundant type of white blood cells and play a key role in the defense against invading pathogens. Neutrophils use a variety of strategies to eliminate invading microbes: (i) microbial uptake followed by intracellular destruction through an array of proteolytic and oxidative enzymes, (ii) degranulation and secretion of antimicrobial factors such as myeloperoxidase (MPO) leading to extracellular destruction of microbes, and (iii) release of NETs with entrapment and non-phagocytic killing of microbes (88, 89). NETs consist of a network of chromatin fibers decorated with antimicrobial proteins such as neutrophil elastase (NE) and MPO to enable the extracellular killing of bacteria or fungi. Interestingly, neutrophils are apparently able to sense the size of microbes and release NETs selectively in response to large pathogens, thereby minimizing the risk of tissue damage associated with the release of NETs (90). Moreover, increasing evidence suggests that the release of NETs might also occur in non-infectious, sterile inflammation, and may contribute to tissue damage (91). For instance, crystals of monosodium urate, the causative agent of gout, were shown to induce release of NETs (92). Cholesterol crystals can also trigger NET formation, leading to priming of macrophages for cytokine release (80). Furthermore, in a very recent study, exposure to high doses of polystyrene nanoparticles and nanodiamonds triggered a “self-limiting” (resolving) NETosis-driven inflammation in mice (93). No NET formation was seen in response to large (100–1,000 nm) particles. We recently observed size-dependent triggering of NETs in primary human neutrophils exposed to GO with a more pronounced effect seen for micrometer-sized GO sheets versus GO sheets with nano-sized lateral dimensions; we also observed a disruption of lipid rafts in neutrophils incubated with GO (Mukherjee et al., unpublished results). Care was taken to control for endotoxin contamination, as LPS is known to prime neutrophils for NET production. Effects of GBMs on neutrophils *in vivo* could impact adversely on the innate immune defense; this remains to be studied.

## Effects on Dendritic Cells: Aiding and Abetting

DCs are professional antigen-presenting cells (94) and as such they are indispensable for the regulation of the balance between immunity (literally meaning “exemption,” the capability of an organism to resist microorganisms) and tolerance (i.e., indifference or non-reactivity toward substances that would otherwise elicit an immune response; an active rather than a passive condition). DCs take up foreign molecules as well as host-derived proteins and process them intracellularly to antigens that are presented in the context of major histocompatibility (MHC) class I and II molecules on the cell surface. In a recent *in vitro* study, pristine GO was found to suppress antigen presentation to T cells using OVA as a model antigen (95). DCs were exposed to GO prior to OVA-loading and then mixed with B3Z86/90.14 (B3Z) CD8+ T cells specific for the H-2K^b^-restricted anti-mouse OVA257-264 (SIINFEKL) peptide. Production of IL-2 was monitored as a sign of T cell activation upon recognition of the OVA epitope 257–264 in the context of the H-2K^b^ molecules (MHC class I). Interestingly, while GO also stimulated maturation of DCs, the immunosuppressive effect of GO was dominant (95). Further studies are needed to understand whether all GBMs behave in this way. In fact, as discussed below, some varieties of GO have shown promise as antigen carriers.

Commonly used adjuvants (i.e., agents that are added to a vaccine to boost the immune response toward a specific antigen) include substances such as mineral oil and alum or other inorganic compounds. However, while these compounds have been in clinical use for many years, the precise mechanism of action remains poorly understood (96). Recent studies showed that the aluminum adjuvant, alum triggered the release of IL-1β in macrophages and DCs in an NLRP3-dependent manner (97), and mice deficient in Nalp3 failed to mount a significant antibody response to an antigen administered with aluminum adjuvants (98). In contrast, Freund’s complete and incomplete adjuvant (i.e., mineral oil with or without inactivated mycobacteria) appeared to act in an inflammasome-independent manner. Sun et al. (99) demonstrated that aluminum-based adjuvants can be engineered to optimize their immunostimulatory properties. Specifically, the authors synthesized a library of aluminum oxyhydroxide (AlOOH) nanorods and compared these to commercial alum and could show that shape, crystallinity, and hydroxyl content played an important role in NLRP3 inflammasome activation (99). Rettig et al. (100) provided evidence that particle size may also influence the immune response to “danger.” Using single-stranded RNA (a known “danger” signal) mixed with protamine to form particles of different sizes, the authors could show that particle size determined whether an anti-viral or anti-bacterial/anti-fungal immune response was triggered. This was suggested to be due at least in part to the selective phagocytosis of nano-sized particles by plasmacytoid DCs, which produced interferon-α. It will be of interest to study the potential effects of GBMs of differing lateral dimensions on DCs and whether these materials could also be exploited as adjuvants to stimulate immune responses. GBMs might also prove advantageous as antigen carriers. Li et al. (101) exploited the fact that GO can spontaneously adsorb proteins to explore the use of this material for intracellular vaccine delivery. Using an *in vitro* model, the authors could show that GO adsorbed proteins were efficiently internalized by DCs leading to antigen cross-presentation to CD8+ T cells. In a more recent *in vivo* study, polymer-modified GO (GO-PEG-PEI) with nano-scale lateral dimensions was shown to act as an antigen carrier to shuttle antigens into DCs (102). Furthermore, compared with free *Helicobacter pylori* Urease B antigen and the clinically approved aluminum adjuvant-based vaccine (Alum-Ure B), GO-PEG-PEI-Ure B was found to induce stronger cellular immunity upon intradermal administration (102). Pristine GO or GO-PEG did not show the same effect. The high surface area of GO allowing for high antigen loading capacity along with the positive charge afforded by the polymer coating could help to explain this effect. The possibility that GO *per se* could have adjuvant properties should also be explored, in light of the fact that small and large GO sheets trigger the NLRP3 inflammasome (discussed above). Finally, Meng et al. (103) recently reported that ultrasmall GO decorated with the antioxidant compound carnosine modulates innate immunity and improves adaptive immunity. The authors could show that GO covalently modified with carnosine, when mixed with the model antigen, OVA promoted robust and durable OVA-specific antibody responses, increased lymphocyte proliferation efficiency, and enhanced CD4+ T and CD8+ T cell activation. The authors proposed that GO-carnosine could be useful as an adjuvant to effectively enhance humoral and innate immune responses *in vivo*.

## Concluding Remarks

In the current essay, we have highlighted recent research on the interactions of GBMs, in particular GO, with the immune system, focusing our discussion mainly on *in vitro* studies. While we are far from a comprehensive understanding of these interactions, one may ask whether there are any general conclusions at this point. One technical, yet non-trivial issue when performing studies of GBMs and immune-competent cells concerns the importance of knowing not only the test material (10), and whether there are traces of endotoxin as this may impact on subsequent immune responses, but also the test system, i.e., the cell model including the composition of the cell medium, and whether this is supplemented or not with serum. Furthermore, it is important to realize that the plasma membrane is not only an impassive barrier between the interior of a cell and the extracellular space but also serves as an important platform for cellular communication between cells, and between the exterior and interior of a cell (104). This is true not least for immune-competent cells that are specialized in sensing and sampling their environment. It follows from this argument that the effects of a biomaterial on the cell membrane could have ramifications for immune cell communication and function. It is of interest to note that the adjuvant, alum, was previously shown to trigger responses in DCs by altering membrane lipid structures, demonstrating that not all immune signaling is receptor mediated, and suggesting that the plasma membrane could behave as a “sensor” for solid structures (105). Thus, the impact of a biomaterial is not necessarily linked to whether or not the material is internalized as direct effects on the plasma membrane could also come into play. In the field of nanotoxicology, much time and effort has been devoted to the determination of the dose of nanoparticles delivered to and internalized by cells, but for atomically thin materials with large lateral dimensions, some toxicological outcomes may depend on direct effects on the plasma membrane, and not only on cellular uptake of the material. In other words, as we continue to probe immunological responses toward GBMs and other 2D materials, we should not forget that significant insights may come from studying seemingly superficial interactions. Or, as actress Ava Gardner once put it, “Deep down, I’m pretty superficial.”

## Author Contributions

BF wrote the manuscript, with input from SPM and MB. The final version of the manuscript was discussed and approved by all participating authors.

## Conflict of Interest Statement

The authors declare that the research was conducted in the absence of any commercial or financial relationships that could be construed as a potential conflict of interest.
